# Monoclonal antibodies against influenza viruses: a clinical trials review

**DOI:** 10.3389/fimmu.2025.1669073

**Published:** 2025-10-10

**Authors:** Kenneth Gabriel Mota, Ana Maria Moro

**Affiliations:** ^1^ Biopharmaceuticals Laboratory, Butantan Institute, São Paulo, Brazil; ^2^ Interunits Graduate Program in Biotechnology, University of São Paulo, São Paulo, Brazil; ^3^ CeRDI – Center for Research and Development in Immunobiologicals, Butantan Institute, São Paulo, Brazil

**Keywords:** influenza, monoclonal antibodies, clinical trials, pandemic virus, antivirals, biopharmaceuticals

## Abstract

The zoonotic influenza viruses cause seasonal epidemics and occasional pandemics, posing a significant public health threat. Transmitted by influenza A and B viruses, they result in ~1 billion annual infections, 3–5 million severe cases, and 300,000–500,000 deaths worldwide, with U.S. healthcare costs reaching $87.1 billion yearly. Understanding viral biology is crucial for developing effective treatment and prevention strategies. This review analyzes 27 clinical trials of anti-influenza monoclonal antibodies (mAbs) from ClinicalTrials.gov, assessing their therapeutic and prophylactic potential. Some mAbs target conserved viral regions (e.g., hemagglutinin stem, M2e protein) for broad-spectrum neutralization. MHAA4549A demonstrated a 97.5% reduction in viral load in H3N2 models and showed synergistic effects with oseltamivir in severe cases. However, despite preclinical promise, others, such as VIR-2482 (intramuscular) and MEDI8852, failed in Phase 2 trials. Safety profiles were generally favorable, with mild Emergent Adverse Events (EAEs) (headache, gastrointestinal disturbances). Key challenges include poor mucosal tissue penetration and variable clinical responses. While mAb-oseltamivir combinations accelerated recovery in hospitalized patients, larger cohorts lacked statistical significance. Viral evolution remains a significant hurdle, emphasizing the need to target conserved epitopes. Future strategies may optimize half-life (e.g., Fc modifications in VIR-2482), improve mucosal delivery, and integrate mAbs with vaccines/antivirals. mAbs hold promise for high-risk groups and pandemics but require further engineering to enhance efficacy and overcome biological barriers. Refinements in administration and design could establish monoclonal antibodies (mAbs) as a key tool in the management of influenza.

## 1 Introduction

Influenza viruses are zoonotic respiratory pathogens that affect humans, primarily caused by influenza A and B viruses. These viruses warrant significant attention as they are responsible for seasonal epidemics and occasional pandemics, posing a substantial threat to global public health. Therefore, understanding the virus’s biology is essential for developing treatment and prevention strategies (1, 2). The World Health Organization (WHO) reports that annual influenza epidemics result in one billion infections, causing 3 to 5 million severe cases and leading to 300,000 to 500,000 deaths worldwide each year. The annual healthcare cost in the United States alone is $87.1 billion (1, 2).

Influenza cases manifest as pandemics, epidemics, outbreaks, and isolated sporadic cases. Seasonal epidemics tend to occur in winter in temperate climates, whereas in tropical regions, they can happen in any season. The epidemiological pattern of the disease directly reflects viral antigenic changes, which continuously generate new strains, alter transmission capacity, and affect population susceptibility (3). Infection can occur at any age; however, the risk of severe complications requiring hospitalization and leading to death is higher among children aged 0 to 2 years, individuals over 65 years old, and pregnant women. In the latter group, the most prevalent complication is pneumonia, which can increase maternal mortality and disability rates (3, 4).

Viral dispersion is highly efficient in human-to-human contact, occurring through respiratory droplets and direct contact. The most common symptoms of infection range from a mild respiratory illness affecting the upper respiratory tract, characterized by fever, sore throat, runny nose, cough, headache, muscle pain, and fatigue. In severe cases, the condition can progress to severe pneumonia and opportunistic bacterial infections in the lower respiratory tract. They can also cause non-respiratory complications, affecting other systems and organs (2).

**Figure 1:**
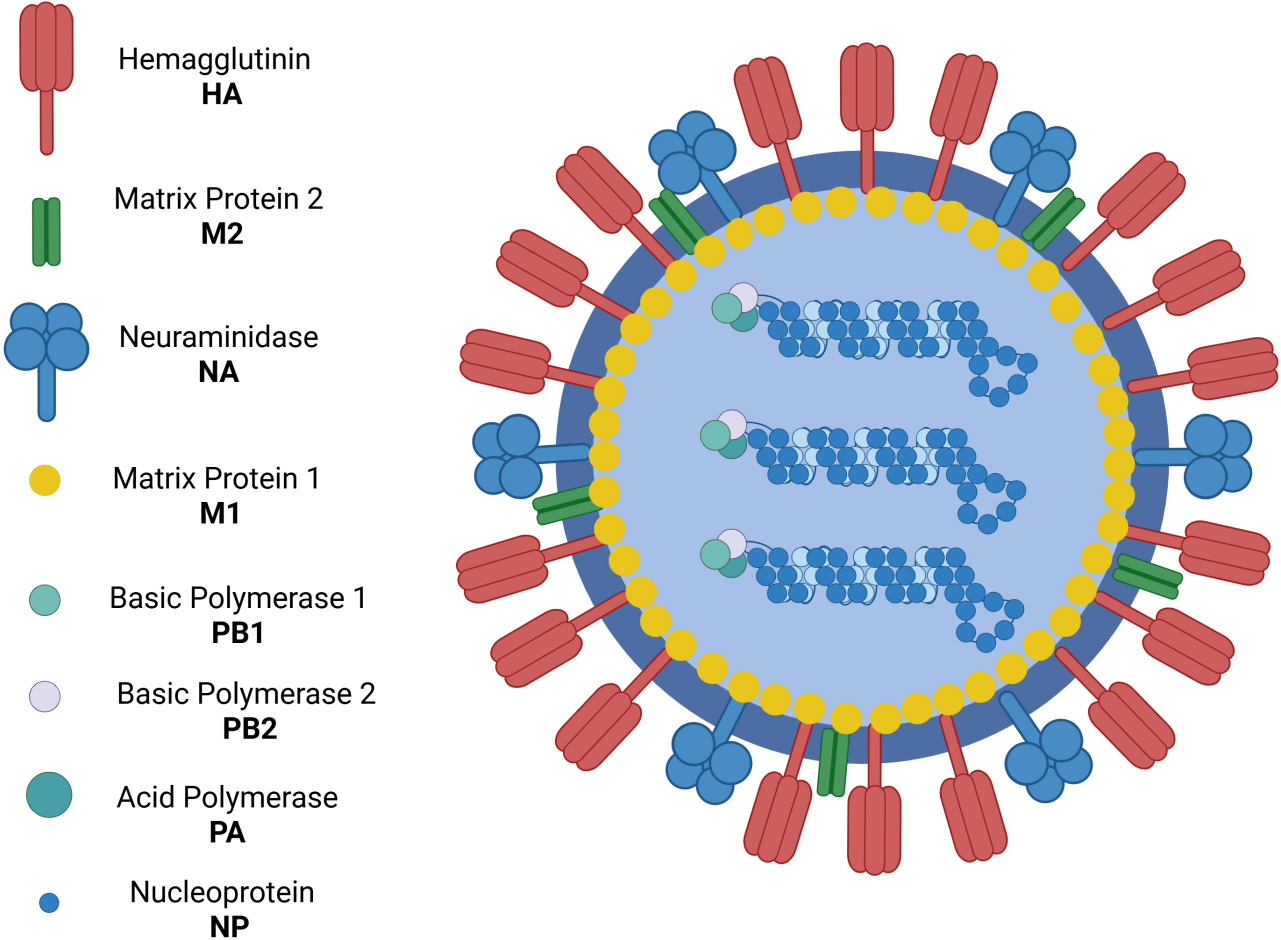
Influenza virus structure. Created with BioRender, 2025.

The Orthomyxoviridae family includes the viruses that cause influenza (5). All influenza viruses are single-stranded, negative-sense RNA viruses with a segmented genome. Influenza A and B viruses contain eight RNA segments that encode two polymerases - one acidic and one basic - as well as virus glycoproteins such as hemagglutinin (HA), responsible for viral entry into the host cell, and neuraminidase (NA), which facilitates the release of new virions from the host cell. Additionally, they encode viral nucleoprotein, the non-structural protein NS1, and the nuclear export protein (NEP) (6) (**Figure 1**).

**Figure 2:**
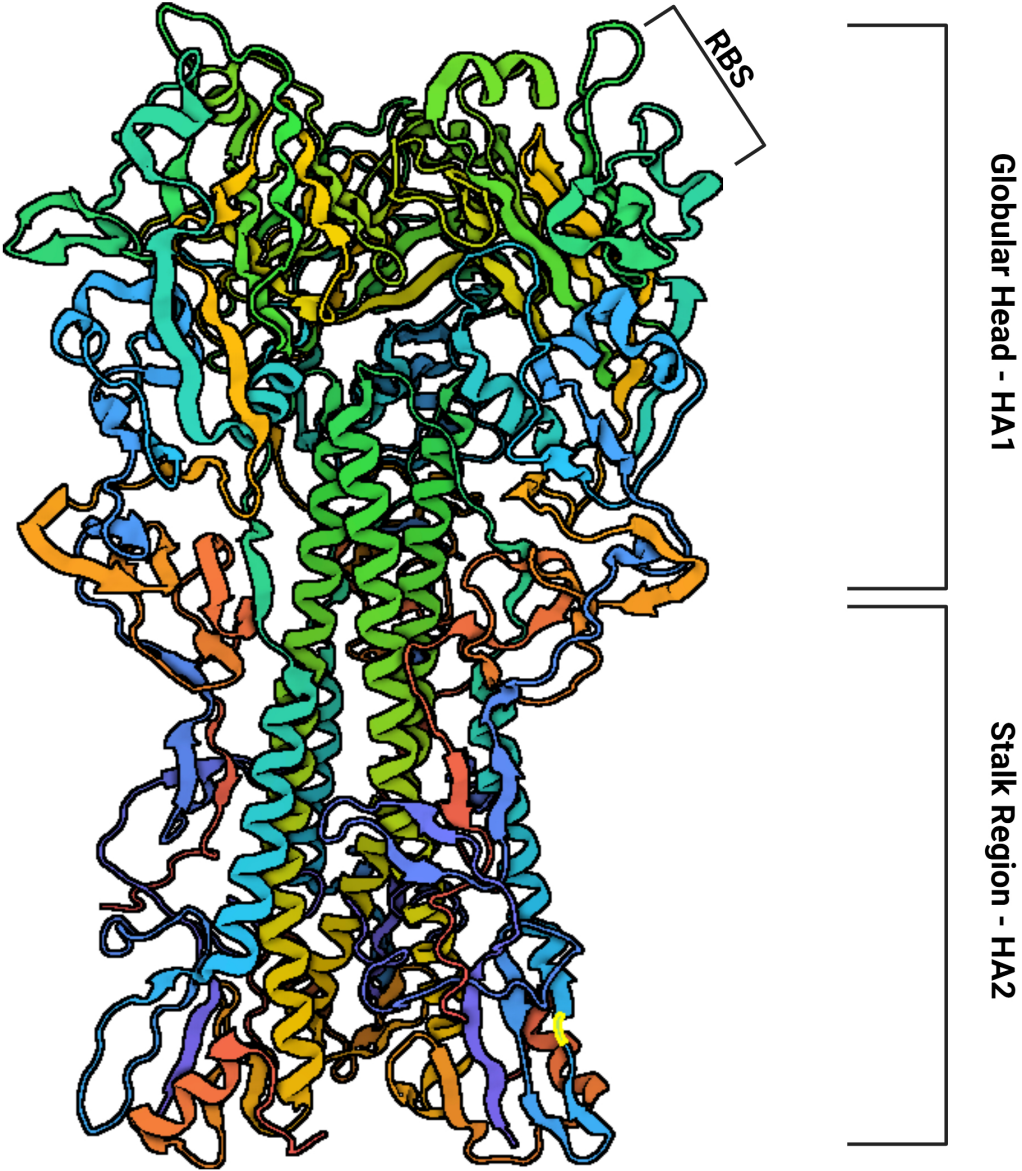
The structure of hemagglutinin is subdivided into a globular head (HA1) containing the Receptor Binding Site (RBS) and a stalk region (HA2). Based on: Protein Data Bank (PDB) Code: 1RU7.

Influenza A viruses are classified into subtypes based on the glycoproteins present on their surface, hemagglutinin (HA) (**Figure 2**) and neuraminidase (NA) (7). There are 17 subtypes of influenza A hemagglutinin (H1–H17), divided into two phylogenetically distinct groups: Group 1 includes H1, H2, H5, H6, H8, H9, H11, H12, H13, H16, and H17, while Group 2 consists of H3, H4, H7, H10, H14, and H15. In contrast, influenza B is divided into only two lineages, Yamagata and Victoria (8).

Influenza A viruses continuously evolve through various evolutionary processes, primarily antigenic drift, which is defined by the gradual accumulation of mutations in viral surface proteins, mainly hemagglutinin (HA) and neuraminidase (NA). This allows the virus to evade host immune recognition. High viral mutation rates drive this process under selective pressure from the host immune system. Studies have shown that substituting a single amino acid near the receptor-binding site in hemagglutinin is sufficient for immune escape. These mutations occur at specific, limited positions in this antigen, suggesting a predictable pattern of antigenic drift (9).

Another evolutionary force is antigenic shift, which involves the introduction of a new viral subtype into human populations. This occurs through genetic reassortment between human and avian influenza viruses. Such events can lead to pandemics and epidemics, as populations have little or no immunity to the newly generated strain. An example was the H1N1 virus, which resulted from a reassortment between avian, swine, and human influenza viruses, causing a global crisis in 2009 (9).

The high mutation rate of the influenza virus presents a significant challenge for prevention strategies. Vaccination is a fundamental method for preventing infections, as it induces antibodies that neutralize the infection. However, due to the virus’s rapid mutation rate, vaccines require constant updates to keep up with viral evolution (9, 10). Vaccine updates occur annually, following the guidelines of the World Health Organization’s (WHO) Global Influenza Programme, which publishes recommendations for vaccine composition for both the Northern and Southern Hemispheres. These recommendations are based on continuous epidemiological monitoring conducted by the WHO (10).

The effectiveness of licensed influenza vaccines varies from year to year, with an estimated efficacy of 40% to 60%, depending on the antigenic match between the strains used in vaccine formulation and the circulating strains. The high variability makes it challenging to prevent seasonal influenza cases. Vaccine efficacy may be even lower in children, the elderly, and immunocompromised individuals (11). There is a search to develop universal vaccines for the influenza virus using various methodologies, which could protect against all strains for a period of more than 1 year, but this is not a reality in the clinic to date (12). Antiviral medications used to treat influenza have limited efficacy against certain strains due to the development of antiviral resistance, highlighting the urgent need for effective tools for influenza treatment. Among the promising alternatives are monoclonal antibodies (mAbs), biopharmaceuticals with the potential for prophylaxis and providing passive immunity, and can also be used as a therapeutic option for ongoing infections. Currently, broadly neutralizing monoclonal antibodies (bnAbs) against influenza A and B are under clinical investigation (13).

**Figure 3:**
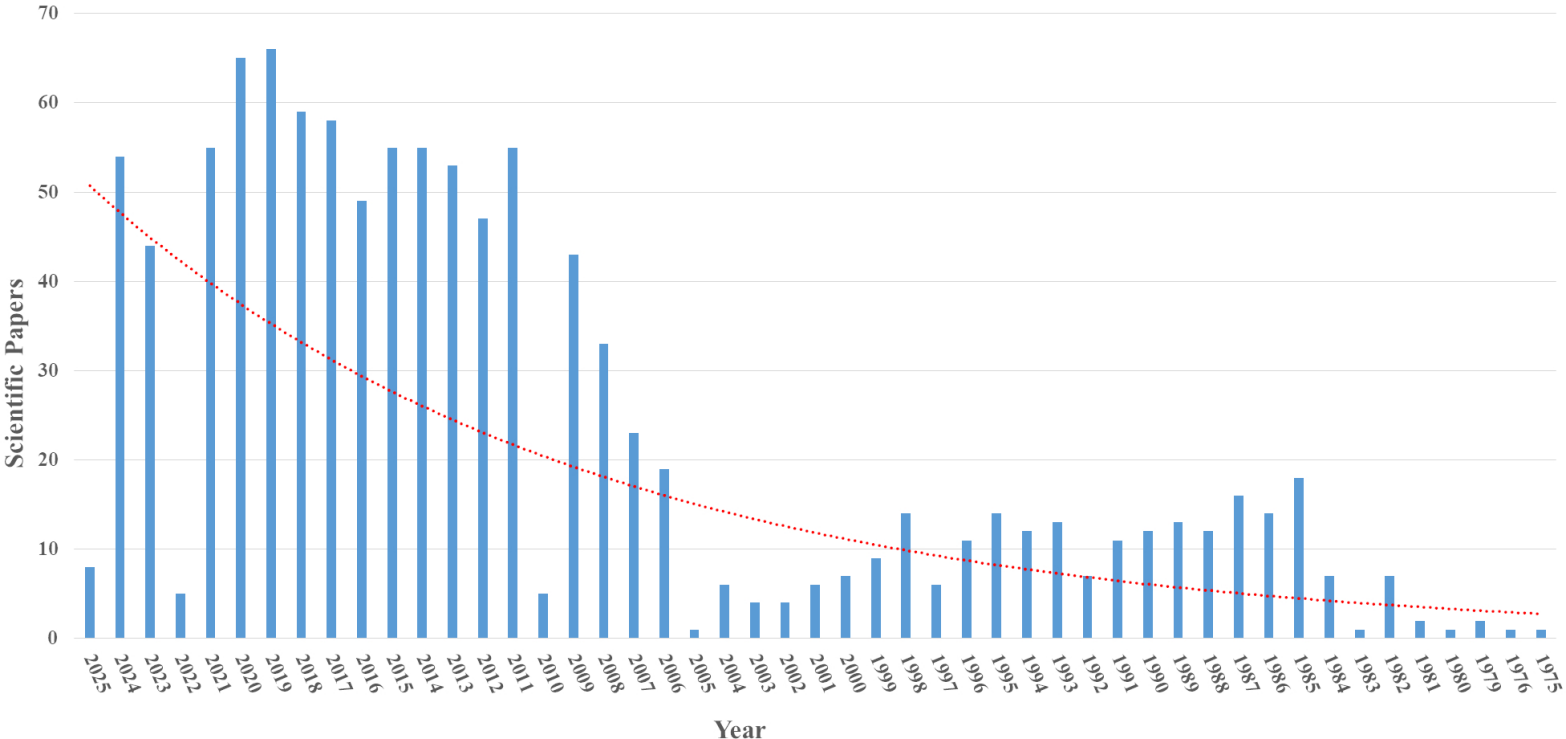
Articles indexed in the PubMed database with the search term “Monoclonal antibodies against influenza virus”.

A search in the PubMed database using the terms “Monoclonal antibodies against influenza virus” identified 1083 scientific articles published between 1975 and 2025. Starting in 2007, there was a more significant increase in publications, which may indicate a growing interest in studies on monoclonal antibodies and the influenza virus, considering its epidemic and pandemic potential (**Figure 3**).

Given the need for antiviral alternatives, mAbs and bnAbs are excellent candidates for prophylaxis and treatment. This review will compile clinical trials of monoclonal antibodies against influenza that have been deposited in the ClinicalTrials database (https://clinicaltrials.gov) to provide an update on this field of study.

## 2 Monoclonal antibodies against influenza in clinical trials

The search terms used in this study for the ClinicalTrials database (https://clinicaltrials.gov) were: Condition/Disease: Influenza (Human) and Intervention/Treatment: Monoclonal Antibody (mAb). The search was conducted between December 2024 and July 2025, and 27 clinical studies were reviewed (**Table 1**). Further details were available for the clinical data of monoclonal antibodies published in scientific articles. For others, no articles were published, and only study abstracts were found in the mentioned databases and included in this review.

**Table 1:** Clinical studies indexed in the clinical trials database were identified using the search terms “Condition/disease: Influenza Human” and “Intervention/treatment: Monoclonal Antibody”.

Monoclonal Antibody	Manufacturer (Latest phase)	Influenza strain	Target	Mechanism	Latest Phase	Clinical studies	Year of last update	Main results
TCN-032	Theraclone Sciences, Inc	A	Matrix Protein 2	Interferes with viral budding, CDC, ADCC	2	NCT01719874 NCT01390025	2012	TCN-032 is a safe, well-tolerated antibody that reduces influenza A symptoms and viral load in clinical studies.
CR6261	NIAID	A	Hemagglutinin Stalk	Neutralization	2	NCT01406418 NCT02371668	2020	It is a safe and well-tolerated antibody, without significant clinical efficacy in reducing influenza symptoms or viral load.
CR8020	Crucell Holland BV	A	Hemagglutinin Stalk	Neutralization	2	NCT01756950 NCT02015533 NCT01938352	2019	Antibody that protected mice; it was safe in phase 1, clinical efficacy results were not published.
CR8020 & CR6261	Crucell Holland BV	A	Hemagglutinin Stalk	Neutralization	2	NCT01992276	2014	Study terminated due to unsatisfactory preliminary efficacy results obtained in an influenza challenge trial, and no clinical data were published.
FGI-101-1A6	Functional Genetics Inc	A	TSG101	Disrupts viral budding	1	NCT01299142	2011	Antibody that blocks the release of virions was well tolerated in a phase 1 study, presented consistent PK, and a long half-life at higher doses.
MHAA4549A	Genentech, Inc.	A	Hemagglutinin Stalk	Neutralization + ADCC	2	NCT01877785 NCT02284607 NCT01980966 NCT02293863 NCT02623322	2019	Broad-spectrum antibody, safe, well-tolerated, with favourable PK, without consistent clinical efficacy against influenza.
MHAB5553A	Genentech, Inc.	B	Hemagglutinin	Neutralization + ADCC	1	NCT02528903	2019	Anti-influenza B antibody, safe, well-tolerated with a long half-life, induces ADCC and presents dose-dependent PK without immunogenicity.
MEDI8852	MedImmune LLC	A	Hemagglutinin Stalk	Neutralization + ADCC	2	NCT02350751 NCT02603952 NCT03028909 NCT03903718	2020	Broad-spectrum human anti-HA antibody, neutralizes groups 1 and 2, safe, well tolerated, with linear PK, long half-life, without significant clinical difference.
VIR-2482	Vir Biotecnologia, Inc.	A	Hemagglutinin Stalk	Prevention, long half-life	2	NCT04033406 NCT05567783	2024	It is a MEDI8852-derived antibody with a long half-life, IM administration, safe and well-tolerated, has not demonstrated significant efficacy in preventing influenza A.
VIS410	Visterra, Inc.	A	Hemagglutinin Stalk	Neutralization + ADCC	2	NCT02045472 NCT02468115 NCT02989194 NCT03040141	2022	Safe and well-tolerated, reduces viral load and elimination time, with ADCC activity and dose-dependent PK.
CT-P27	Celltrion	A	Hemagglutinin Stalk	Neutralization + ADCC	2	NCT02071914 NCT03511066	2016	Combination of two anti-HA mAbs (CT149 and CT120), safe in clinical trials, with neutralizing activity, ADCC and therapeutic potential, results not yet published.

### 2.1 TCN-032

TCN-032 is a fully human mAb that targets the ectodomain of the matrix protein 2 (M2e) of influenza A virus, a highly conserved protein. The epitope to which TCN-032 binds is located between amino acid residues 1–9 and 239-252, being present in approximately 99.8% of the influenza A strains reported in humans, birds, and swine (14, 15). *In vivo* studies in mice revealed improved survival with the administration of this antibody against both seasonal and highly pathogenic strains. The benefit of using TCN-032 combined with oseltamivir in this animal model was also observed (14). TCN-032 does not block viral entry into the host cell or inhibit the protein’s function as a proton pump. However, it binds to M2e expressed on the surface of infected cells, reducing viral replication by directly interfering with the budding of new virions, by Complement-Dependent Cytotoxicity (CDC), or Antibody-Dependent Cellular Cytotoxicity (ADCC) (14).

In a Phase 1 study (NCT01390025) conducted by Theraclone Sciences, Inc., which started in 2012 and was titled “Safety Study of Anti-Influenza Virus mAb to Treat Influenza”, the current status is completed, and the last update was in 2012. The study aimed to compare the safety profile in healthy volunteers with the administration of a single escalating dose of TCN-032 (1, 3, 10, 20, or 40 mg/kg) via intravenous infusion. Injection of TCN-032 was well tolerated, with mild to moderate intensity EAE unrelated to the drug under study. The half-life of TCN-032 was estimated to be 15 days, and no immunogenicity was observed (14).

In a Phase 2 study (NCT01719874) conducted by Theraclone Sciences, Inc. in 2012, titled “Influenza Virus Challenge Study to Test mAb TCN-032 as a Treatment for Influenza”, with the current status unknown and the last update from 2012, the goal was to determine the safety and efficacy of TCN-032 in a controlled influenza infection challenge in humans with the influenza A/H3N2 (Wisconsin/67/2005) strain. Twenty-four hours after infection, TCN-032 was administered via intravenous infusion as a single dose of 40 mg/kg or placebo. Patients began treatment with oseltamivir for 5 days, starting on the seventh day post-infection. Although different by 13%, the percentage of 60 participants (29 × 31 in drug and placebo groups, respectively) with any influenza symptoms or fever between days 1 and 7 was similar between the TCN-032 group (35%) and the placebo group (48%) (p = 0.14) The study also measured the effect of TCN-032 compared to placebo on the total influenza symptom score as measured by the area under the curve (AUC, days 1–7). Patients treated with TCN-032 showed a 35% reduction in the median AUC of total symptoms (p = 0.047) and a 2.2 log reduction in the median AUC of viral load, as analyzed by qPCR (p = 0.09), compared to the placebo group (14). Only 2 of 48 positive subjects for influenza infection, both in the placebo group, had pyrexia. PK (pharmacokinetics) reached 16 days, and no immunogenicity was observed in the serum samples. The proportion of mild to moderate EAE was similar for subjects treated with TCN-032 or placebo (14).

### 2.2 CR6261

CR6261 is a broadly neutralizing human antibody against influenza group 1, obtained through phage display technology. It binds to the stalk of hemagglutinin, centered on the HA2 helix A, a highly conserved region (15, 16). In a Phase 1 study (NCT01406418) conducted by Crucell Holland BV starting in 2013, titled “Assessment of CR6261, a mAb Against the Influenza A Virus”, with completed status and the last update from 2013, the objective was to test in healthy individuals the tolerability, PK, safety, and immunogenicity of single and escalating doses of this mAb, with doses ranging from 2 mg/kg to 50 mg/kg, in a double-blind, placebo-controlled study. However, no results were released.

In the Phase 2 study (NCT02371668) conducted by the National Institute of Allergy and Infectious Diseases (NIAID), which started in 2015, titled “Efficacy and Safety of CR6261 in an H1N1 Influenza Healthy Volunteer Human Challenge Model (CR6261)”, the current status is completed, and the last update was in 2020. The objective of the study was to evaluate the efficacy and safety of CR6261 in an H1N1pdm09 infection challenge in healthy individuals, aged 18 to 45 years, non-smokers, unvaccinated against influenza in the previous vaccination season, with an antibody titer measured by the hemagglutination inhibition assay (HAI) ≤1:10. This was a randomized, placebo-controlled, double-blind study. Fifty mg/kg of CR6261 was administered intravenously 24 hours after viral inoculation via the intranasal route. Forty-nine individuals received CR6261, and 42 received a placebo. No statistically significant effect was observed between the two groups (AUC: 48.56 log [copies/mL] × days, interquartile range [IQR]: 202 versus AUC: 25.53 log [copies/mL] × days, IQR: 155, P = 0.315), nor was there a significant clinical effect measured by the Patient-Reported Outcomes Influenza Questionnaire (FLU-PRO), which tracks the symptoms reported by the volunteers. Regarding PK, in the treated group, serum levels reached an average concentration of 1 × 10^6^ ng/mL 15 minutes after infusion, with a progressive decrease over the week, remaining around 3 × 10^5^ ng/mL after 7 days and returning to near-baseline levels by day 66. In nasal swabs, maximum concentration peaks were reached between days 2 and 3, with an average of 597 ng/mL, indicating low penetration into the respiratory mucosa. Shedding in a range of 0 to 9 days occurred similarly between the two groups. Regarding safety, CR6261 was well tolerated by participants, with mild EAE, except for two severe EAE reactions, including urticaria, which interrupted the infusion in these cases and were possibly related to the lot preparation, which was subsequently withdrawn; no infusion reactions were observed when using the other CR6261 lot preparation (16).

### 2.3 CR8020

The mAb CR8020 is a human antibody selected by isolating memory B cells from patients who had been vaccinated against influenza. It targets a highly.

conserved epitope at the base of the HA stalk, being widely neutralizing against group two influenza viruses. In a mouse lethality test with H3N2 or H7N7 infections, a 3 mg/kg dose of CR8020 demonstrated protection for infected animals (17). In a Phase 1 clinical trial of CR8020 (NCT01756950) reported by Crucell Holland BV, initiated in 2013, titled “Assessment of CR8020, a mAb Against Influenza A Viruses”, the status is completed, and the last update was in 2013. The study aimed to assess parameters such as the safety, tolerability, PK, and immunogenicity of single and escalating doses of CR8020 in healthy individuals. The study was randomized, double-blind, and placebo-controlled with dose escalation from 2 mg/kg to 50 mg/kg. No results have been published.

In the Phase 2 study (NCT01938352), also conducted by Crucell Holland BV, initiated in 2013, titled “Evaluation of the Protective Efficacy and Safety of CR8020 in an Influenza Challenge”, its current status is completed, and the last update was in 2019. The study aimed to evaluate the protective efficacy and safety of CR8020 in a human influenza challenge. The study was a randomized, double-blind, placebo-controlled trial. Healthy individuals of both sexes, aged >18 and <45 years, received a prophylactic intravenous infusion of 15 mg/kg followed by a challenge with the H3N2 virus (unreported strain). The study, with its primary outcome of evaluating viral load at the nasopharyngeal mucosa, was completed in 2019; however, no results were published.

Another Phase 1 study (NCT02015533) conducted by Janssen Pharmaceutical, initiated in 2013, titled “A Study to Assess the Safety, PK, and Immunogenicity of CR8020 in Japanese Healthy Participants”, currently has the status Withdrawn (The study stopped early, before enrolling its first participant), with its last update in 2019. The study aimed to assess the product’s safety, PK, and immunogenicity in a randomized, double-blind, and placebo-controlled study, where a single dose of CR8020 (50 mg/kg) would be administered by intravenous infusion to healthy Japanese male participants. The individuals would be followed for up to 99 days, but the study was terminated before any participants were recruited.

### 2.4 CR8020 and CR6261

A Phase 2 study (NCT01992276) conducted by Crucell Holland BV in the United States, initiated in 2013, titled “Assessment of Efficacy of CR8020 and CR6261, Monoclonal Antibodies, Against Influenza Infection”, its current status is withdrawn (The study stopped early, before enrolling its first participant), and the last update was in 2014. The study evaluated whether CR8020 or CR6261 could reduce the viral load in hospitalized patients with confirmed influenza A infection. It was conducted as a randomized, double-blind, placebo-controlled trial, with patients receiving standard therapy for influenza A infection. A cohort of 262 patients was planned to receive 30 mg/kg of CR8020, 30 mg/kg of CR6261, or placebo via intravenous infusion, with a duration of 117 days for each participant. At this point, the incidence of EAE or serious EAE, as well as survival times, would be reported. After hospital discharge, participants would be followed up on outpatient visits. The study was planned to be held in 70 locations across 12 countries. The results were not published, and the study was withdrawn due to preliminary efficacy results from an influenza challenge trial in 2014.

### 2.5 FGI-101-1A6

The anti-TSG101 antibody, unlike most monoclonal antibodies developed for influenza, has a different target. Instead of binding to a viral epitope, it binds to a cellular marker, the product of the tumor susceptibility gene 101 (TSG101). This is a highly conserved epitope that becomes exposed on the membrane of influenza-infected cells. In healthy cells, the protein remains intracellular. The matrix protein M1 of the influenza virus directly interacts with the UEV (Ubiquitin E2 Variant) domain of TSG101, causing TSG101 to be exposed on the cell membrane and facilitating the budding of new influenza virions. By blocking TSG101, the release of new virions is interrupted (18). FGI-101-1A6 is a human monoclonal anti-TSG101 antibody. In the Phase 1 study (NCT01299142) conducted by Functional Genetics Inc., initiated in 2011, titled “Safety and PK Study of Human mAb (FGI-101-1A6)”, the current status is unknown, with the last update in 2011. The study aimed to determine the safety and tolerability of the anti-TSG101 administered intravenously in healthy volunteers aged 18–45 years in a single dose. Secondary outcomes were PK and immunogenicity evaluation. The study was placebo-controlled, and no results have been published to date. A report dated March 2013 by Leyla Diaz (ADA607997) is available (19). In this phase 1a clinical study in healthy volunteers, with six ascending dose cohorts, starting from a minimum dose of 0.0017 mg/kg up to 10 mg/kg, with the primary objective of assessing the safety and tolerability of the drug, and as a secondary objective to evaluate its PK. In this study, the results obtained with higher doses were more consistent in terms of PK. In the same group of higher doses (1.5 mg/kg, 5.0 mg/kg, and 10.0 mg/kg), the mean estimates of Cmax and AUC increased with the increasing dose, but not proportionally, with an average half-life of 170 to 287 hours. The results presented in the study indicate that mAb FGI-101-1A6, administered as an intravenous infusion in healthy adult volunteers, was well tolerated at all doses and was not associated with any local irritation.

### 2.6 MHAA4549A

The monoclonal MHAA4549A, initially published by Nakamura et al. in 2013 (20) under the identity 39.29, is a fully human antibody derived from sorted B cells. It binds to a highly conserved region in the stalk of hemagglutinin, with neutralizing capacity for both 1 and 2 groups of influenza virus, including H1, H2, H3, H5, and H7 variants., acting by two complementary mechanisms: avoiding hemagglutin-mediated membrane fusion by binding to hemagglutinin on viral particles and exbiting ADCC by binding to HA on the surface of infected cells (21). Genentech, Inc. conducted two Phase 1 studies. The first (NCT01877785), initiated in 2013, titled “A Study of MHAA4549A to Assess Safety and PK in Healthy Volunteers”, was completed, with the last update in 2016. The study aimed to evaluate the safety, efficacy, tolerability, and PK of MHAA4549A in 21 healthy volunteers. The study was a randomized, double-blind, placebo-controlled trial with a single ascending dose of 1.5, 5, 15, and 45 mg/kg (four individuals in each dose, plus six receiving placebo). Considering safety, 61.9% of the volunteers experienced EAE, most of which was classified as mild, with headaches being the most common (25%). In terms of PK, the maximum concentration (Cmax) increased proportionally with the applied dose, ranging from 33.5 µg/mL (1.5 mg/kg) to 1180 µg/mL (45 mg/kg), and the mean life ranged from 21.9 to 24.6 days (21).

In 2014, Genentech, Inc. indexed another Phase 1 study in the ClinicalTrials database (NCT02284607), titled “A Study of High Dose MHAA4549A in Healthy Volunteers”. The current status is completed, with the last update in 2017. The objective of the study was to evaluate the safety, tolerability, and PK of two fixed high single intravenous doses of 8400 mg (dose 1) or 10800 mg (dose 2) (approximately 135 mg/kg) in 14 healthy volunteers randomized into two cohorts of 4 volunteers to receive dose 1 or dose 2, or 6 volunteers receiving a placebo. Mild EAE was reported in 85.7% of the volunteers, the most common being headache (50%) and nasopharyngitis (38%). Regarding PK, the mean half-life of MHAA4549A was 21.5 days, with Cmax of 3570 µg/mL (8400 mg) and 4780 µg/mL (10800 mg). Immunogenicity tests were conducted in both trials, and no volunteer showed antibodies against MHAA4549A (21).

In the Phase 2 study (NCT01980966) conducted by Genentech, Inc., which started in 2013, titled “A Study of MHAA4549A in Healthy Volunteers in an Influenza Challenge Model”, with the current status marked as completed and the last update in 2017, the study aimed to evaluate the safety and efficacy of MHAA4549A in an influenza challenge model. It was a double-blind, randomized, placebo-controlled study involving 101 healthy adult volunteers aged 18 to 45 years, seronegative for A/Wisconsin/67/2005 (H3N2), as measured by the hemagglutination inhibition (HAI) assay. This strain was used for the challenge. After viral inoculation, individuals were randomly assigned to receive a single intravenous dose of MHAA4549A at 400, 1200, or 3600 mg, or a placebo, 24 to 36 hours after infection by intranasal inoculation with 50% tissue culture infectious particles. Of those who received a placebo, eight were selected to receive a standard dose of oseltamivir 24 to 36 hours after viral inoculation (22). Safety, PK, and immunogenicity were evaluated up to 120 days. The participants were assessed for viral load, influenza symptoms, and inflammatory biomarkers. In the treated group compared to the placebo, individuals who received 3600 mg of MHAA4549A showed a 97.5% reduction in viral load by AUC, as measured by qPCR (11 log10 viral copies/mL.h, while the placebo had 458 log10 viral copies/mL.h (p = 0.005). Those who received 1200 mg showed a reduction of only 3% (444 log10 viral copies/mL.h, p = 0.902), and those who received 400 mg showed a 46% reduction (247 log10 viral copies/mL.h, p = 0.046). The group receiving oseltamivir showed an 87% reduction compared to the placebo (57 log10 viral copies/mL.h, p = 0.059). The group receiving 3600 mg showed statistically significant decreases in overall viral burden and peak viral load. The viral shedding was also reduced. All three doses were considered safe, with symptoms related to influenza infection (total mucus, fever, and inflammatory cytokines) reduced in the 3600 mg-treated group. There was no treatment effect in the 1200 mg group. PK were consistent with those observed in Phase I clinical trials. No immunogenicity was detected (22).

Genentech, Inc. conducted another Phase 2b study (NCT02293863) in 2015 titled “A Study of MHAA4549A in Combination with Oseltamivir Versus Oseltamivir in Participants with Severe Influenza A Infection”. The current status is completed, and the last update was in 2018. The study aimed primarily to shorten the median time to normalization of respiratory function by removing patients from oxygen supplementation or mechanical ventilation, thereby maintaining a stable saturation of 95%. It also aimed to evaluate the safety, PK, and viral load due to a single intravenous dose of MHAA4549A in hospitalized adults with severe influenza A confirmed by a rapid test or PCR. The study enrolled 166 patients in 18 countries, was double-blind, placebo-controlled, and divided into three cohorts: (i) Placebo + oseltamivir = 56; (ii) 3600 mg MHAA4549A + oseltamivir = 55; (iii) 8400 mg MHAA4549A + oseltamivir = 47 (8 patients were removed from the study due to technical issues). The mAb MHAA4549A was administered as a single intravenous dose, in comparison to a placebo, in combination with oral oseltamivir at 75 mg or 150 mg twice daily for at least 5 days. The primary endpoint, reduction in the time to normalization of respiratory function (SpO2 > 95%), showed no significant results. The group receiving 8400 mg MHAA4549A in combination with oseltamivir had an average time of 2.65 days to normalize respiratory function, while the oseltamivir-only group took 4.28 days. The group that received 3600 mg MHAA4549A plus oseltamivir took 2.78 days. The treated groups did not show a reduction in viral load or improved clinical outcomes. EAE was similar for the groups. The 30-day mortality rates were 9.1% in the 8400 mg MHAA4549A group, 7.7% in the 3600 mg MHAA4549A group, and 5.6% in the placebo group with oseltamivir monotherapy. As for PK, the half-life of MHAA4549A was approximately 17–19 days. Regarding immunogenicity, only 1.3% of patients developed anti-MHAA4549A antibodies (23). In another Phase 2 study (NCT02623322) conducted by Genentech, Inc., starting in 2016, titled “A Study of MHAA4549A as Monotherapy for Acute Uncomplicated Seasonal Influenza A in Otherwise Healthy Adults”, with the current status of completed and the last update in 2019, the objective was to evaluate the safety, tolerability, efficacy, and PK of a single dose of 3600 mg or 8400 mg of MHAA4549A intravenously in adults aged 18–65 years with acute uncomplicated seasonal influenza A, confirmed by rapid test or PCR no later than 72h after symptoms onset. This was a randomized, double-blind, placebo-controlled clinical study conducted at 35 sites across six countries. The cohort comprised the randomization og 124 patients, with 43 receiving a placebo, 41 receiving 3600 mg of MHAA4549A, and 40 receiving 8400 mg of MHAA4549A. Regarding EAE, the frequency was similar between the treated group (33.1%) and the placebo group (30.2%), with nausea (6.5%) and bronchitis (4%) being the most common symptoms. Bronchitis events, occurring in 4 of the treated group and 1 of the placebo group, were considered mild to moderate and unrelated to the drug treatment. No severe EAE cases were reported. Regarding symptom relief time, the median was 154 hours (3600 mg), 146 hours (8400 mg), and 117 hours (placebo), with no significant difference (HR: 0.92 and 0.90; 80% CI). Regarding viral load, there was no statistically significant difference between the treated and placebo groups. Regarding PK, the Cmax was 1050 ± 299 µg/mL (3600 mg) and 2190 ± 58 µg/mL (8400 mg). No hospitalizations, influenza reinfections, or deaths were reported up to 100 days after evaluation. The drug was well-tolerated, but no clinical efficacy was observed (24).

### 2.7 MHAB5553A

MHAB5553A, a fully human mAb of the IgG1 type derived from plasmablasts of vaccinated donors, is directed against a conserved epitope in the esterase domain of the influenza B virus hemagglutinin, neutralizing Victoria and Yamagata strains by binding to the hemagglutinin on the virus and the membrane of infected cells, inducing ADCC (25). In a Phase 1 study (NCT02528903) conducted by Genentech, Inc., starting in 2015, titled “A Study to Investigate the Safety, Tolerability, and PK of MHAB5553A in Healthy Volunteers”, with the current status of completed and the last update in 2019, the aim was to investigate the safety, tolerability, and PK of MHAB5553A in healthy volunteers. The study was a randomized, double-blind, placebo-controlled, dose-escalating trial. It included 26 volunteers aged 18–65 who received a single ascending intravenous dose of MHAB5553A. The doses administered were 120 mg, 1200 mg, 3600 mg, 8400 mg, or 10800 mg, randomized in a 4:1 ratio (4 individuals received treatment for every one that received a placebo), except for the 120 mg cohort, where the ratio was 4:2. Regarding EAE, 84.6% of participants reported some EAE, with 94.5% considered mild. The most frequent events were nasopharyngitis (57.7%), headache (34.6%), and elevated creatine phosphokinase marker (7.7%). Regarding PK, the half-life was approximately 19–20 days, with Cmax serum ranging from 40.9 µg/mL (120 mg) to 5260 µg/mL (10800 mg) in a dose-dependent manner. The Cmax nasal ranged from 1.46 µg/mL (120 mg) to 278 µg/mL (10800 mg) and was nonlinear and non-dose proportional. Immunogenicity testing showed no detection of anti-MHAB5553A antibodies in the volunteers’ serum. The drug was well-tolerated, with no signs of dose-dependent toxicity (25).

### 2.8 MEDI8852

MEDI8852, developed by MedImmune (AstraZeneca), is a broadly neutralizing IgG1 kappa mAb that can recognize both group 1 and 2 influenza A strains. It binds to the center of HA2 helix A, a highly conserved region on the hemagglutinin stalk, preventing its cleavage and consequently blocking the initiation of the infectious process. A first antibody derived from a donor, along with its clonally related sequences, was reconstructed to a common, non-mutated ancestral sequence, demonstrating neutralization of Group 1 influenza strains. This antibody was further modified by point mutagenesis to generate MEDI8852 mAb, which can neutralize both Group 1 and Group 2 strains, as well as more than 80 years of influenza antigenic evolution (26). Neutralization by MEDI8852 occurs at the beginning of the infection by inhibiting HA-mediated membrane fusion, and also at the end of the infection cycle, it can prevent the formation and spread of new infective particles, besides binding to HAs on the membrane of infected cells, recruiting NK (natural killer) cells, macrophages, and complement for cytotoxicity. In preclinical trials, a lethal influenza challenge was conducted in mice using the H5N1 and H7N9 strains. MEDI8852 was tested alone or in combination with oseltamivir as a therapeutic measure. A similar study challenged ferrets with H5N1 or H7N9 viruses, treating the animals with either MEDI8852 alone or combined with oseltamivir. Both studies concluded that MEDI8852 alone was more effective than oseltamivir alone in preventing animal death, reducing fever, and alleviating overall clinical symptoms. The antibody was capable of blocking influenza transmission in ferrets. Combining the mAb with oseltamivir provided the highest efficacy (27). MEDI8852 advanced to phase 1 clinical trials (NCT02350751) conducted by MedImmune LLC, initiated in 2015, titled “Phase 1 Placebo-controlled, Dose-escalation Study to Evaluate the Safety and PK of MEDI8852 in Adults (MEDI8852)”. Its current status is completed, and the last update was in 2015. The study aimed to evaluate the safety, PK, and immunogenicity of the drug. The study was double-blind, placebo-controlled, and single-dose escalation conducted in healthy adult individuals. It included 40 volunteers, of whom 32 received MEDI8852 and 8 received a placebo. The participants were randomized into four cohorts, receiving doses of 250 mg, 750 mg, 1500 mg, or 3000 mg of MEDI8852 in a 3:1 or 5:1 ratio. No severe EAEs were observed, and their incidence was similar between the treatment group (37.5%) and the placebo group (37.5%). The most commonly reported EAE was headache. As for PK, it was determined to be linear, increasing proportionally with the dose administered, with an average half-life of ~19.4 to 22.6 days. No volunteer developed anti-drug antibodies (ADA) during the 100-day evaluation period (28).

In the phase 2a study (NCT02603952) with MEDI8852 conducted by MedImmune LLC, initiated in 2015, titled “A Phase 2a Study to Evaluate the Safety of MEDI8852 in Adults with Uncomplicated Influenza (MEDI8852)”, its current status is completed, and the last update was in 2018. The study’s objective was to evaluate the safety and tolerability of a single intravenous dose of MEDI8852 in combination with oseltamivir, as well as both drugs separately, in adult participants with uncomplicated acute influenza A confirmed by a rapid test. The study was randomized and partially double-blinded. A total of 126 participants aged 18 to 65 in United States and South Africa were randomized into four cohorts: cohort 1 (n = 31) received 750 mg of MEDI8852 in combination with 75 mg of oseltamivir, cohort 2 (n = 31) received 3000 mg of MEDI8852 and 75 mg of oseltamivir, cohort 3 (n = 32) received placebo and a 75 mg dose of oseltamivir, and cohort 4 (n = 32) received only 3000 mg of MEDI8852. The patients were monitored for influenza symptoms, EAE, and viral clearance. As for safety and tolerability results, the EAE rate was 41.9% in the MEDI8852-treated group and 31.3% in the oseltamivir-only group. The most common EAE was bronchitis, occurring in 11.8% of the MEDI8852 group and 3.1% of the oseltamivir group, followed by pharyngitis in balanced proportions of 3.2 and 3.1, respectively. Of all EAE, severity grade 3 was observed in 3 volunteers receiving the high-dose of MEDI8852 in combination with oseltamivir and 2 receiving placebo plus oseltamivir. One infusion-related reaction was attributed to the high-dose antibody plus oseltamivir. All other events were considered mild or moderate. Regarding viral load reduction, it reached undetectable levels by day 5 of treatment (log_10_ 3.1 copies/ml) across all cohorts, indicating no statistically significant difference in viral load reduction between the cohorts. The virus titer was available for 11 subjects, and was not detected in 7 out of 9. One subject had a decrease in virus titer by day 5, followed by an increase on days 7 and 9. In terms of PK, the results were linear, with serum levels proportional to the administered dose, ranging from 131 µg/mL (750 mg MEDI8852 + Oseltamivir) to 619 µg/mL (3000 mg MEDI8852 + Oseltamivir) and 652 µg/mL (3000 mg MEDI8852 in monotherapy). The time to resolution of symptoms was similar between the groups, with medians ranging from ~95 hours (placebo plus oseltamivir) to 188 hours (MEDI8852 alone), and overlapping confidence intervals (29).

There were two additional Phase 2 studies indexed in the ClinicalTrials database, both conducted by MedImmune LLC with MEDI8852. One study from 2017 (NCT03028909) was titled “Dose Ranging Study to Evaluate the Efficacy and Safety of MEDI8852 in Adults Who Are Hospitalized with Type A Influenza”. Still, its current status is “Withdrawn”, meaning the study was prematurely terminated before enrolling participants, and its last update was in 2019. The second study, initiated in 2020 (NCT03903718), was titled “Evaluation of the Safety and Efficacy of a mAb for Treating Influenza”, also with a status of Withdrawn.

### 2.9 VIR-2482

The mAb VIR-2482 is derived from MEDI8852 by the introduction of M428L/N434S (LS) mutations, which confer recirculation-mediated half-life extension through the neonatal Fc receptor FcRn. The modification was planned to allow its administration once per flu season. Another modification to the formulation, to 150 mg/mL, allowed for its administration intramuscularly (IM) rather than intravenously (IV), enabling its use in an outpatient setting. Based on the failure of previous clinical trials to confer efficacy when the antibody was administered after symptom onset, after days of virus circulation, the VIR-2482 antibody was designed for passive immunization for the prevention of seasonal influenza, applied to individuals not protected by vaccination and those who cannot be vaccinated, including an influenza pandemic situation (30).

Phase 1 study (NCT04033406) conducted by Vir Biotechnology, Inc. in Australia, initiated in 2019, titled “Study of VIR-2482 in Healthy Volunteers”, has been completed, with the last update in 2022. The study aimed to evaluate the safety, tolerability (primary endpoints), PK, and immunogenicity (secondary endpoints) of VIR-2482 in healthy adults. It was a randomized, double-blind, placebo-controlled study involving 100 healthy participants aged 18-65, allocated into four dose cohorts. Cohort 1, with 20 participants, received 300 mg of VIR-2482, and 5 participants received a placebo, administered via a 2mL IM injection. Cohort 2 consisted of 1200 mg of VIR-2482 for 20 participants, with 5 participants receiving a placebo, administered as two 2mL IM injections. Cohort 3 consisted of 1800 mg of VIR-2482 in 20 participants; 5 received a placebo, fractionated into three 4mL IM injections. Cohort 4 consisted of 60 mg of VIR-2482 in 20 participants; 5 received a placebo, administered via a 0.4mL IM injection. The injection site was the gluteal region. Regarding safety and tolerability, VIR-2482 was well tolerated across all doses administered. EAE occurred in 68.8% of the treated group and 85% of the placebo group, with most EAE being mild to moderate. The most commonly reported adverse events were headache, cough, and upper respiratory tract infection. Injection site reactions were mild and occurred in only 7.5% of participants in the treated group. Regarding PK, the average time to reach maximum concentration (Tmax) was 7 days for the higher doses and 12.5 days for the 60 mg dose. The half-life ranged from 56.7 to 70.6 days, allowing for single-dose administration per flu season. The mucosal passage of VIR-2482 (exploratory endpoint) may be limited, as analyses showed that only 2-5% of the serum concentration reaches the upper respiratory tract mucosa. The incidence of ADA was considered low, affecting 8% (6/80) of all participants, of which 5 had pre-existing ADA; transient ADA formation was detected in one of the participants with only a low titer (1:8). Overall, the evidence of ADA did not affect the mAb’s PK or its safety (30).

Phase 2 study (NCT05567783) conducted by Vir Biotechnology, Inc., initiated in 2022, titled “A Phase 2 Study to Evaluate the Efficacy and Safety of VIR-2482 for the Prevention of Illness Due to Influenza A”, has been terminated (the study has stopped early and will not restart). Participants are no longer being examined or treated, with the last update in 2024. The study aimed to evaluate the safety and efficacy of VIR-2482 in preventing influenza A in healthy, unvaccinated adults. It was a randomized, double-blind, placebo-controlled clinical study during the 2022–2023 flu season in the United States, enrolling 2,977 healthy, unvaccinated participants, randomized into three groups: one receiving 450 mg of VIR-2482, the second receiving 1200 mg of VIR-2482, and the third group receiving a placebo, all via IM. The study aimed to assess whether VIR-2482 reduced the incidence of influenza-like symptoms, evaluated by the Influenza-Like Illness (ILI) instrument and confirmed by RT-PCR. However, none of the doses significantly reduced the risk of contracting influenza. The relative risk reduction (RRR) for the 450 mg dose was only 3.8% (95% CI: -67.3% to 44.6%), while for the 1200 mg dose, the RRR was 15.9% (95% CI: -49.3% to 52.3%). Secondary efficacy endpoints followed the Centers for Disease Control and Prevention (CDC) definition: fever >37.8 °C, cough or sore throat, and RT-PCR positive for influenza A, as well as the World Health Organization (WHO) definition: fever >38 °C, cough, and RT-PCR positive for influenza A. VIR-2482 was expected to prevent and reduce the appearance of these symptoms in the treated groups. In the 1200 mg group, there was a reduction of 57.2% (95% CI: -2.5% to 82.2%) according to the CDC definition and 44.1% (95% CI: -50.5% to 79.3%) according to the WHO definition. In *post-hoc* analyses, excluding results occurring from the first 7 days after administration, the better results suggest that the antibody may need more time to reach effective levels (31). Regarding safety and tolerability, VIR-2482 was well tolerated, with EAE similar between the groups. The most commonly reported reactions were upper respiratory tract infections, sore throats, cough, and muscle aches (myalgia). Injection site reactions were mild and transient. As for PK, the Tmax was reached in 6.95 days for the 1200mg dose, with a half-life ranging from 54.7 to 55.4 days. The drug concentrations in serum were similar between infected and non-infected participants, indicating that the study’s failure was not due to low drug exposure. One participant had a greater than 4-fold increase in ADA titer relative to baseline. Despite the drug being safe and well-tolerated there was no evidence of its clinical efficacy according to the endpoints. (31).

### 2.10 VIS410

VIS410 is a human mAb of the IgG1 type that binds to the hemagglutinin stem, with enhanced specificity and affinity by the approach of atomic interaction network analysis that introduced changes in the CDR and FWR of an existing antibody scaffold. It targets a constrained epitope on HA binding all HAs with high avidity in both the virus and virus-infected cells (32–34). In a phase 1 study (NCT02045472) conducted by Visterra, Inc., in the United States, initiated in 2014, titled “A Study of VIS410 to Assess Safety and PK”. Its current status is completed, and the last update was in 2015. The study primarily evaluated the safety and tolerability of single escalating doses of VIS410 in healthy volunteers. It was a Phase 1, double-blind, placebo-controlled study involving 41 healthy volunteers, divided into five cohorts of 4 participants each, who received single doses of VIS410 at 2, 5, 15, 30, and 50 mg/kg. Additionally, 11 participants across the cohorts received a placebo. The results showed that VIS410 was well tolerated at the evaluated doses, with EAE classified as mild to moderate in 65.9% of participants in the treated group. In comparison, 63.3% of those in the placebo group reported similar events. Nervous system disorders represented the most prevalent EAE at similar proportions across all cohorts, followed by gastrointestinal disorders, reported only for the VIS410 group (10 of 30 subjects and 5 of 6 in the 50 mg/kg VIS410 cohort). Infections and infestations occurred similarly between the VIS410 and placebo cohorts. Testing of nasopharyngeal swabs of individuals who develop clinically significant upper respiratory infections was negative for influenza up to 120 days (35). Regarding PK (secondary endpoint), the drug had an average half-life in the serum of 12.9 days, and its maximum concentration in the upper respiratory tract was 25.3 µg/mL and 1316 µg/mL in the serum of patients who received a dose of 50 mg/kg. Immunogenicity was also evaluated, none at baseline, resulting in 4 out of 30 participants developing ADA, not altering drug PK. (35).

Phase 2a study (NCT02468115) conducted by Visterra, Inc., in Belgium, initiated in 2015, titled “Influenza Challenge Study of VIS410 in Healthy Volunteers”. Its current status is completed, and the last update was in 2016. The study aimed to evaluate the safety profile and the effect of VIS410 administered by intravenous infusion 24 hours after a 10^6^ TCID50 of A/California/7/2009 H1N1 influenza infection challenge administered intranasally in healthy individuals aged 18 to 45. The study was divided into three parts: one was randomized, double-blind, and placebo-controlled, with 31 participants receiving a dose of 2300 mg of antibody or placebo in a 7:5 ratio. The second and third parts were open-label, with 11 and 4 participants receiving 2300 mg or 4600 mg of VIS 410, respectively. Low antibody titers (Hemagglutination assay - HAI ≤10) against the challenge strain were considered for volunteer selection (36). VIS410 demonstrated a significant reduction in viral load, with a 76% decrease in the area under the curve (AUC) of viral load measured by qRT-PCR (p = 0.024) and a 91% decrease in the AUC in viral culture (TCID50, p = 0.019), accompanied by lower viral load peaks compared to the placebo group. Consistently lower virus shedding was demonstrated by qRT-PCR and TCID50 for all VIS410-treated arms compared to placebo in all parts of the study. Clinical symptoms were mild to moderate, with similar resolution between groups, but a tendency for a faster resolution of upper respiratory tract symptoms in the VIS410-treated group (36). Regarding safety and tolerability, EAEs were reported in 97% and 77% of the VIS410-treated and placebo groups, respectively.

VIS410 was associated with gastrointestinal events, including abdominal pain and diarrhea, which were observed to be linked to a transient increase in cytokine profiles (IL-8, TNF-α, and, to a lesser extent, IL-6). These levels returned to baseline between 48 and 60 hours. EAE was considered mild to moderate, albeit four VIS410 recipients experienced severe cramping, diarrhea, or both. Cytotoxicity evaluation showed a substantial increase in ADCC activity in VIS410-treated individuals with two H7N9 strains (36).

Phase 2a study (NCT02989194) conducted by Visterra, Inc., in five countries, initiated in 2017, titled “Study of an Investigational mAb, VIS410, in Subjects With Uncomplicated Influenza A”, its current status is completed, and the last update was in 2022. The study aimed to evaluate the safety and tolerability of the mAb VIS410 in individuals with uncomplicated influenza A virus infection. It was a randomized, double-blind, placebo-controlled study where 148 participants, confirmed with influenza A and with symptoms onset within a maximum of 72 hours, aged between 18 and 65, were randomized in a 1:1:1 ratio (46 patients received a high dose of 4000 mg intravenously, 44 received a low dose of 2000 mg, and 48 received a placebo). All participants were pre-treated with diphenhydramine, combined with either ibuprofen or aspirin, to mitigate gastrointestinal effects of EAE. The rates of EAE were dose-dependent, with 55% of patients treated with VIS410 at 4000 mg, 35% in the VIS410 at 2000 mg group, and 24% in the placebo group, indicating a statistically significant difference between the high-dose group and the placebo group. The most common EAE events reported were diarrhea, vomiting, and headache, all of which were classified as mild and self-limiting. Three severe grade events occurred, one episode of gastritis in the VIS410–4000 mg group and two others in the placebo group. Through the FLU-PRO questionnaire, symptom evaluation revealed faster symptom improvement in the group treated with VIS410, particularly in the 2000 mg group, with an average time to symptom reduction of 2.1 days compared to 2.6 days in the placebo group; however, this difference was not statistically significant (p = 0.173). Viral shedding was significantly (p = 0.03) reduced to 1.9 days compared to 3.6 days in the placebo group. (37). Antiviral activity testing, measured by TCID50 culture of material collected via nasopharyngeal swab, showed a reduction in AUC up to day 7 of infection, with a lower median of 3.66 in the VIS410-treated group compared to 4.78 in the placebo group (p = 0.08). qRT-PCR analyses did not reveal significant differences between the treated and control groups, which may be attributed to the technique’s inability to distinguish between neutralized and active virus. In PK evaluations, VIS410 showed a dose-proportional half-life of approximately 10 days, with penetration into the nasal cavity of 3-4% of serum levels. In immunogenicity evaluations, 23% of recipients developed ADA with minimal impact on PK parameters. An analysis of HA sequencing in 107 paired samples (baseline versus post-treatment) presented 15 amino acid mutations, with 6 in each VIS410 group and 3 in the placebo group (37).

A phase 2b study (NCT03040141) was conducted by Visterra, Inc., initiated in 2018, titled “Study of Efficacy and Safety of IV VIS410 Plus Oseltamivir Versus Oseltamivir in Hospitalized Adults with Influenza A”. Its current status is completed, and the last update was in 2022. The study aimed to compare the efficacy of VIS410 in combination with oseltamivir versus oseltamivir alone in hospitalized adults with severe cases of Influenza A on oxygen support. The last update was in 2022; however, the data is not yet available.

### 2.11 CT-P27

CT-P27 is a mixture of two monoclonal antibodies (CT149 and CT120) in a 1:1 combination to cover neutralization of both groups 1 and 2 of the influenza viruses. Both antibodies bind to the hemagglutinin stem in close, slightly overlapping epitopes, without interference. In animal studies, CT149 was found to protect mice from H1N1, H3N2, and H5N1 infections; however, it did not efficiently neutralize group 1 viruses *in vitro*. CT120 was selected for its efficient neutralization of group 1 viruses.

In a mouse model of infection, CT-P27 significantly reduced mortality and viral load. Combination with oseltamivir increased the survival rate of infected mice. In addition to its neutralizing potential, CT-P27 demonstrated the induction of ADCC and inhibition of new viral particle release from infected cells (38).

A phase 2a study (NCT02071914), conducted by Celltrion with CT-P27, initiated in 2014, titled “A Study to Evaluate the Efficacy and Safety of CT-P27 in an Influenza Challenge Model”, has a current status of completed, with its last update in 2020. The study aimed to evaluate the safety and efficacy in an influenza challenge model and assess whether there was a reduction in viral load, as measured by qRT-PCR, in the nasopharyngeal mucosa. It was a randomized, double-blind, placebo-controlled study in which enrolled individuals received a single intravenous dose of 10 mg/kg CT-P27, 20 mg/kg CT-P27, or a placebo. Despite the study’s completion, the results have not yet been published.

In 2022, Celltrion conducted a phase 2b study (NCT03511066), initiated in 2016, titled “A Study to Evaluate the Efficacy and Safety of CT-P27 in Acute Uncomplicated Influenza A Infection”. Its current status is terminated (The study has stopped early and will not reopen; and participants are no longer being examined or treated). The last update was in 2022. The study aimed to evaluate the efficacy and safety of CT-P27 in acute, uncomplicated influenza A infection. The study was a double-blind, randomized, placebo-controlled trial with a single dose of 90 mg/kg CT-P27, 45 mg/kg CT-P27, or placebo administered intravenously. The study results have not been published to date.

## 3 Discussion

Influenza remains a significant public health challenge due to its high morbidity and mortality and the virus’s ability to mutate rapidly, limiting the effectiveness of seasonal vaccines. Monoclonal antibodies represent a promising therapeutic and prophylactic tool against the influenza virus. This review analyzed 27 clinical trials indexed in the ClinicalTrials database, highlighting the successes and challenges encountered in the research. Antibodies like MHAA4549A significantly reduced viral load (97.5% in AUC in H3N2 challenge models) and showed synergistic effects with oseltamivir in severe cases. Although MHAA4549A reduced viral load, the clinical benefits were not statistically significant, which could be attributed to the study being conducted in young, healthy volunteers who developed only mild and self-limiting illnesses, thereby limiting the ability to detect symptomatic differences. In addition, there was variability in infection and pre-existing immunity, and antibody exposure in the nasal mucosa was shown to be non-linear. Some individuals, despite achieving a good virological response, did not achieve sufficient antibodies in the mucosa to improve their symptoms. Finally, the study was primarily designed to assess virological outcome and had limited statistical power to detect minor clinical differences, which may have contributed to the lack of significance (22).

In contrast, others, such as VIR-2482 and MEDI8852, showed mixed results, failing to achieve favorable outcomes in phase 2 trials despite favorable preclinical data. The efficacy of VIR-2482 may have been hampered by an early influenza season that year, which allowed infections to occur before participants could benefit from treatment. Additionally, a poorly sensitive primary endpoint and the lag between serum and tissue concentrations in IM delivery may have contributed. Viral resistance and manufacturing or PK failures were ruled out. The 1200 mg dose may have reduced influenza A illness, as suggested by secondary endpoints (31). Several mAbs displayed broadly neutralizing potential in preclinical tests; however, there was significant variability in their clinical efficacy, influenced by factors such as dosage and administration routes. The mAb therapy is sought to neutralize the virus, which causes infection days before the onset of symptoms. In animal models, it is easier to manage the timing of infection and antibody administration, as well as the administration route, which is usually intraperitoneal. Despite the high value of preclinical testing, the limitations should be considered. Mice are preferred due to their low cost, high reproductive rate, and ease of manipulation. However, the pathogenesis of influenza viruses is not completely replicated in mice. Except for highly pathogenic strains, mice are not naturally infected with influenza viruses and display different symptoms when challenged. Ferrets are more attractive as an animal model for influenza infection, as they are naturally and highly susceptible to the virus, and their clinical symptoms are similar to those in humans. Their high cost, size, lack of specific reagents, and requirement for high-level safety laboratories are drawbacks that limit the use of ferrets (39, 40). Only one mAb (MEDI8852) was reported to be tested in ferrets (27). In infectious disease models, viral antigens may mutate differently in animals, leading to overestimation of mAb efficacy compared to the human context (41).

All the studies analyzed regarding the safety profile showed favorable results, generally reporting mild EAE. Gastrointestinal adverse reactions were reported to VIS410 mAb, with diarrhea being the most common (24.4%); cases of moderate diarrhea were accompanied by nausea and vomiting at the highest dose (50 mg/kg) reported in 5 out of 6 participants, being two classified as Grade 2 EAE, and observed to be linked to cytokine release (36). The reactions were resolved by the end of the study without withdrawal of consent. The authors suggest that these gastrointestinal symptoms may be linked to infusion reactions and that adjusting infusion rates or using premedication could help mitigate them in future studies (35).

To reduce the occurrence of EAE related to mAbs, different combined strategies can be adopted, ranging from molecule engineering, with humanization, use of fully human mAbs, glycoengineering, and formulations that reduce aggregates to minimize immunogenicity, to clinical management, which includes pre-medication with antihistamines and corticosteroids, slow and monitored infusions, and dose adjustment according to response and toxicity. Equally important are pre-treatment screening and identification of latent infectious diseases and comorbidities, with continuous monitoring of laboratory parameters and the occurrence of ADA (42). The administration route can influence injection site reactions; while IV injections are given slowly, IM injections depend on highly concentrated mAb formulations. Safety is of utmost importance when mAbs are used as prophylaxis, particularly in vulnerable populations.

Typically, mAb administration strategies involved IV infusion, except for VIR-2482, which was formulated for IM administration, offering logistical advantages but not translating into significant clinical protection in Phase 2 trials. Combination therapies, particularly those involving oseltamivir, showed potential for accelerating recovery in hospitalized patients; however, this effect was not statistically significant in larger cohorts. A key limitation in mAb treatment resembles what occurs with vaccines: the rapid evolution of viruses. Thus, focusing on conserved regions of the virus in mAb development may overcome this limitation. An alternative approach involves the development of monoclonal antibodies targeting neuraminidase, as proposed by Momont et al., who developed an mAb capable of blocking the enzyme’s activity and classified it as pan-neutralizing against influenza (43).

Challenges related to the low availability of mAbs in mucosal tissues have been identified in some studies. Future efforts may prioritize optimizing antibody design for prolonged half-life (e.g., Fc modifications in VIR-2482) and improving delivery in mucosal tissues. The integration of antibody structure with existing antivirals or vaccines can maximize efficacy, as in the case of the drug CD388, which consists of a human IgG1 Fc domain modified to increase the molecule’s half-life, conjugated to a zanamivir dimer. This neuraminidase inhibitor simultaneously binds to multiple active sites of the enzyme, leading to viral aggregation and blocking the release of new viral particles. With its extended half-life, prophylactic use becomes feasible, making it a potent neutralizer against several influenza A and B strains, maintaining effectiveness even against zanamivir-resistant strains. CD388 was tested in mice and macaques, yielding results that combine the high potency and broad spectrum of zanamivir with the prolonged half-life of a monoclonal antibody, providing universal, durable, and potent protection against influenza A and B in preclinical testing (44).

Integrating mAbs with existing antivirals or vaccines may maximize efficacy, although further investigation is required. In conclusion, while mAbs hold potential as a valuable tool for managing influenza, particularly in more vulnerable or immunocompromised individuals or during pandemic scenarios, their full potential depends on overcoming some biological barriers posed by the virus itself. Antibodies have longer half-lives than small molecules, allowing for less frequent dosing and sustained protection. Innovative approaches, such as nasal administration, would lead to a more effective clinical capacity. Antibodies stand out for their broad neutralization capacity, ability to engage multiple immune mechanisms, and reduced risk of resistance, making them a promising and versatile tool for treating influenza, especially as viral diversity and drug resistance challenge therapies (13). Strategic refinements in antibody engineering, composition, administration, and combination regimens are essential to establish mAbs as a valuable tool against the evolving threat of influenza.
